# A real – life observational pilot study to evaluate the effects of two-week treatment with montelukast in patients with chronic cough

**DOI:** 10.1186/1745-9974-10-2

**Published:** 2014-03-20

**Authors:** Roxana K Mincheva, Tanya Z Kralimarkova, Miroslava Rasheva, Zlatko Dimitrov, Denislava Nedeva, Maria Staevska, Vera Papochieva, Penka Perenovska, Karina Bacheva, Vasil D Dimitrov, Todor A Popov

**Affiliations:** 1 Clinical centre of Allergy and Asthma, Alexander’s University Hospital, 1 Georgi Sofyiski Str, 1431 Sofia, Bulgaria; 2 Clinic of Paediatric Pulmonology, Alexander’s University Hospital, 1 Georgi Sofyiski Str, Sofia, Bulgaria; 3 Clinic & Diagnostic Centre KariLab, 60 Mur Str, Sofia, Bulgaria

**Keywords:** Chronic cough, Cough threshold, Montelukast, Markers of inflammation, Exhaled breath temperature

## Abstract

**Background:**

Different conditions make the proximal airways susceptible to tussigenic stimuli in the chronic cough (CC) syndrome. Leukotrienes can be implicated in the inflammatory mechanism at play in it. Montelukast is a selective cysteinyl-leukotriene receptor antagonist with proven effectiveness in patients with asthma. The aim of our real-life pilot study was to use montelukast to relieve cough symptoms in patients with CC allegedly due to the two frequent causes other than asthma – upper airway cough syndrome and gastroesophageal reflux (GER).

**Methods:**

14 consecutive patients with CC were evaluated before and after 2 weeks of treatment with montelukast 10 mg daily. Cough was assessed by validated cough questionnaire. Questionnaires regarding the presence of gastroesophageal reflux were also completed. Cough reflex sensitivity to incremental doubling concentrations of citric acid and capsaicin was measured. Lung function, airway hyperresponsiveness and exhaled breath temperature (EBT), a non-invasive marker of lower airway inflammation, were evaluated to exclude asthma as an underlying cause. Thorough upper-airway examination was also conducted. Cell counts, eosinophil cationic protein (ECP), lactoferrin, myeloperoxidase (MPO) were determined in blood to assess systemic inflammation.

**Results:**

Discomfort due to cough was significantly reduced after treatment (P < 0.001). Cough threshold for capsaicin increased significantly (P = 0.001) but not for citric acid. The values of lactoferrin and ECP were significantly reduced, but those of MPO rose. EBT and pulmonary function were not significantly affected by the treatment.

**Conclusion:**

Patients with CC due to upper airway cough syndrome or gastroesophageal reflux (GER) but not asthma reported significant relief of their symptoms after two weeks of treatment with montelukast. ECP, lactoferrin, MPO altered significantly, highlighting their role in the pathological mechanisms in CC. Clinical trial ID at Clinicaltrials.gov is NCT01754220.

## Background

Chronic cough (CC) is typically defined as cough that persists for longer than 8 weeks and its management presents a challenge for the clinician. Ruling out a plethora of less frequent pathologies, three conditions remain that account for 92% of CC in immunocompetent, nonsmoking subjects: 1) upper airway cough syndrome (UACS), also referred to as postnasal drip syndrome, 2) asthma and 3) gastroesophageal reflux (GER) [1,2]. Cough may be a prominent symptom of asthma and management according to the Global Initiative of Asthma (GINA) guidelines usually suppresses it. Similarly, diagnosis and treatment of UACS and GER would in most cases alleviate or abolish bothersome cough.

The underling mechanisms of cough have been extensively investigated. The afferent part of the cough reflex consists of mainly two types of receptors: predominant rapidly adapting receptors (RARs) that respond primarily to mechanical and acidic stimuli (stretch, hypotonic and hypertonic saline, and citric acid) and non-myelinated C-fibers that characteristically respond to chemical and inflammatory stimuli such as histamine, prostaglandins, substance P and capsaicin [3,4]. Citric acid is the most widely used acid tussigen, which works upon sensory fast-conducting nerve endings as well as nociceptors and A-delta fibers [5]. Capsaicin is known to induce cough in a reproducible and dose-dependent way and acts mainly via transient receptor potential vanilloid receptors [6]. Both types of receptors seem to be at play in subjects with CC, their relative involvement shaping the individual pattern of the condition.

Cough receptors are triggered differently in CC pathogenesis. In UACS secretions containing inflammatory mediators are thought to stimulate proximal airway receptors inducing cough. This umbrella term includes sinusitis, allergic and non-allergic rhinitis, (postinfectious rhinitis, rhinitis medicamentosa, vasomotor rhinitis, rhinitis due to physical or chemical irritants) [3]. The exact mechanisms for cough attributable to GER are still debated but the most probable ones are on one hand distal esophageal acid exposure that stimulates an esophageal-tracheobronchial cough reflex via the branches of the vagus nerve, and microaspiration of esophageal contents into the laryngopharynx and trachea eliciting also symptoms like dysphonia and bitter taste [7,8]. Furthermore, esophageal dysmotility occurs with ensuing dysregulation of the aerodigestive reflexes [9].

Cysteinyl - leukotrienes (CysLTs) LTC_4_, LTD_4_ and LTE_4_ are produced from arachidonic acid through the 5-lipoxygenase pathway within inflammatory and structural cells, including mast cells, eosinophils, basophils, dendritic cells, lymphocytes, bronchial epithelial and smooth muscle cells [10]. They exert their proinflammatory, bronchoconstrictive and mucosecretory effects through interaction with their receptors, CysLT_1_R and CysLT_2_R [11]. The possible mechanism of CysLTs in CC could be direct since it has been shown that CysLTs stimulate the release of substance P and other tachykinins [12], and indirect, by exerting their bronchoconstrictive effects or inducing secretions from the inflamed tissues affecting both types of cough receptors. Montelukast ingested orally is a selective and potent cysteinyl - leukotriene receptor antagonist (CysLTRA) that exerts its action by blocking CysLT_1_Rs. It has already passed the scrutiny of many elaborate randomized controlled trials and its efficacy and safety in treating patients with asthma have been proven. LTRAs are now on the list of drugs recommended for asthma treatment by the Global Initiative for Asthma guidelines (GINA) [13]. It has also been speculated that the range of action of montelukast stretches out in any type of inflammatory process in which leukotrienes are involved, acting also in a CysLT_1_R-independent manner [14]. Its effectiveness in rhinitis has been shown [15], but there is paucity of data about the other conditions underlying CC [16,17]. Nevertheless, randomized controlled trials, although recognized as the ‘gold standard’ for establishing efficacy, operate in an idealized environment and can only measure efficacy in limited, artificially selected populations. Thus, our idea was to interfere as little as possible in the average patients’ population that comes to see the doctor and to observe what effects montelukast might have in patients with CC which is not due to asthma.

In this pilot study we wanted to explore in real life settings the effectiveness of two weeks treatment with montelukast in patients with chronic cough on the patients’ perception of discomfort due to cough coupled with measurement of cough threshold to recognized tussigenic stimuli. Moreover, we wanted to see whether changes in local airway and systemic inflammatory markers paralleled the subjective effects of montelukast treatment.

## Methods

### Study design and patients

The study was conducted as a pilot observational real life trial. Fourteen consecutive out-patients referred to the Clinic of Allergy and Asthma in Sofia, Bulgaria were enrolled. They had subjective and objective measurements taken at the beginning and after two weeks treatment period with montelukast (Alvokast, Alvogen Pharma Bulgaria) 10 mg in the evening. We have chosen two weeks as an arbitrary duration of treatment shortening the 4 week period that other investigators used for patients with cough-variant asthma [18,19]. Patients ranged between 15 and 69 years in age, 9 were women and all met the inclusion criterion of having cough for 8 weeks or longer and the exclusion criteria of current use of ACE-inhibitors, use of systemic and inhaled steroids in the last 4 weeks, concomitant severe disease, COPD, pregnancy and current smoking. All patients had normal chest radiographs. They underwent standard examinations to substantiate inflammatory changes in the oral and nasal cavities, assessments of their lung function, airway responsiveness and atopic state. Based on the results from this diagnostic algorithm we excluded patients in the spectrum of asthmatic conditions. All of the patients signed an informed consent. The study was approved by the local Ethics committee of Alexander’s University Hospital in Sofia.

Patients were asked to fill in a self-administered validated questionnaire on the impact of cough on quality of life, a modified Bulgarian version of Leicester Cough Questionnaire (LCQ) [20] on both visits and only on the first visit they were to fill in the Frequency Scale for the Symptoms of GER (FSSG) questionnaire to account for the possibility of GER playing a role in their cough complaints [21]. Scores above 8 coupled with complaints of heartburn, dysphagia, bitter taste and dysphonia were rather suggestive of concomitant reflux pathology in 5 of the patients enrolled in the study. Although the use of objective tests for verification of GER complaints is reasonable, till present day there is no unanimous view on this in regard to cough assessment and their routine use in investigation of GER cannot be recommended [22].

### Measurements

Pulmonary function tests were performed (Schiller Spirovit SP – 10 Spirometer) and showed normal volume and dynamic lung parameters (Table 1) with negative bronchodilator/bronchoconstrictor response. Tests aimed at characterizing the cough reflex and airway and systemic inflammation were carried out before onset and after of the two weeks montelukast treatment following the same sequence. Exhaled breath temperature (EBT), a surrogate marker of airway inflammation, was assessed with portable breath thermometer (X-Halo, Delmedica Investments LTD, Singapore) according to a standardized procedure [23]. The evaluation of cough threshold to the tussigenic substances citric acid and capsaicin was done with due resemblance to ERS guidelines on the assessment of cough [24]. In brief, six serial dilutions were attained before every measurement from prepared stock solutions of citric acid 1000 mmol/l and capsaicin 100 μmol/l. For the delivery of the tussigenic substances we used Omron MicroAir portable ultrasonic nebulizer and the patients were asked to take five consecutive tidal breaths of a given concentration. The patients were blinded for the order of delivery and the concentration of capsaicin or citric acid that produced two or five coughs (C_2_ and C_5_ respectively) was recorded. For economic reasons and for the convenience of our out-patients both cough threshold measurements were performed on the same day, although we tried to leave the maximal possible interval between them to minimize the effect of tachyphylaxis [25]. Although current unanimity on C_2_ or C_5_ as the preferred end-point has not been reached, most of the published studies often report both values, but not infrequently C_5_ alone is reported. For the sake of facilitating the statistical analysis we equalized the concentrations with indices (from 1 being the highest dilution to 7 being the stock concentration). The systemic markers of inflammation were high sensitivity C-reactive protein (hsCRP), eosinophil cationic protein (ECP), lactoferrin (LF) and myeloperoxidase (MPO). High sensitivity C-reactive protein (hsCRP) was assessed in a centralized laboratory using latex immunoturbidimetric method (GIESSE Diagnostics s.r.l., Rome, Italy). ECP was measured by sandwich ELISA ECP using a reagent of RADIM S.p.A., Roma, Italy. Lactoferrin and MPO were evaluated by means of immunoenzymatic test using reagents of AESKU Diagnostics, Wendelsheim, Germany.

**Table 1:** Characteristics of the patients completing the study

**Characteristics of patients (n = 14)**	**Variables format and values**
Age [years]	Mean = 43, Standard deviation = 17
Gender [number (%)]	Females = 9 (64), Males = 5 (36)
Atopy [number (%)]	3 (14)
Total IgE [IU/mL]	Median = 69.9, Range = 28.2 ÷ 96.6
Rhinitis symptoms [number (%)]	12 (86)
GER [number (%)]	5 (36)
Duration of cough (weeks)	Mean = 27, Range = 8 ÷ 72
LCQ score before treatment	Mean = 12.4, Standard deviation = 3.36
FSSG score	Mean = 9.9, Standard deviation = 7.8
FVC% predicted before treatment	Mean = 110.6, Standard deviation = 19.9
FEV1% predicted before treatment	Mean = 111.2, Standard deviation = 19.2
EBT before treatment	Mean = 34.46, Standard deviation = 0.54

### Statistical analysis

Demographic data were analyzed using standard descriptive analysis. All numerical data sets were checked for normality by using the Kolmogorov-Smirnov test to allow further processing by parametric or non-parametric tests. Pre-/post- treatment comparisons and pretreatment correlations were sought. P < 0.05 was accepted for two-tailed significance. SPSS 16 software package was used for the analysis.

## Results

The characteristics of the patients are shown on Table 1.

**Figure 1:**
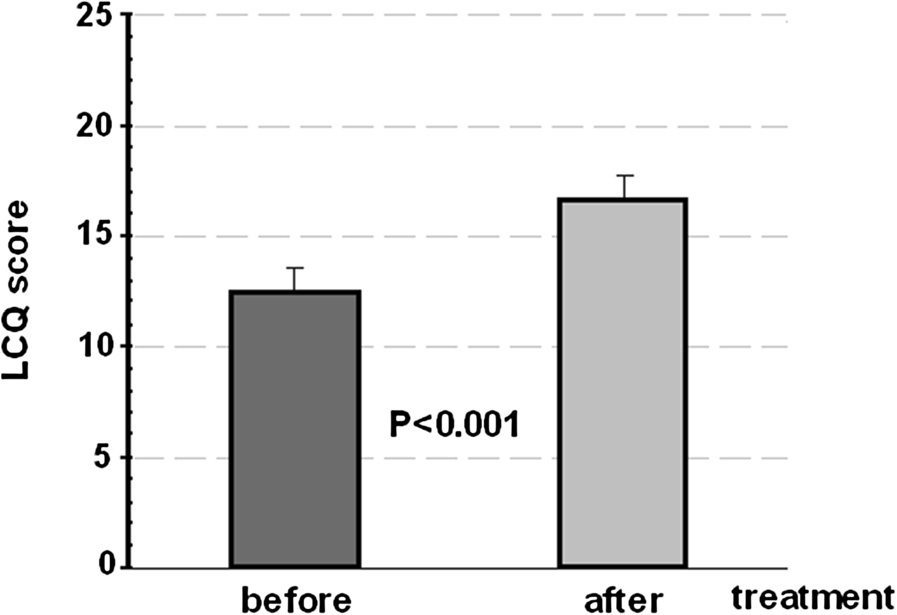
Leicester cough Questionnaire (LCQ) scores before and after treatment.

None of them reported any side effect attributable to the drug. Subjectively all of them had complete or almost complete resolution of the cough complaints. The total LCQ score showed significant improvement from 12.4 (mean) ± 3.4 (standard deviation) to 16.6 ± 3.1 score, P < 0.001 (Figure 1).

**Figure 2:**
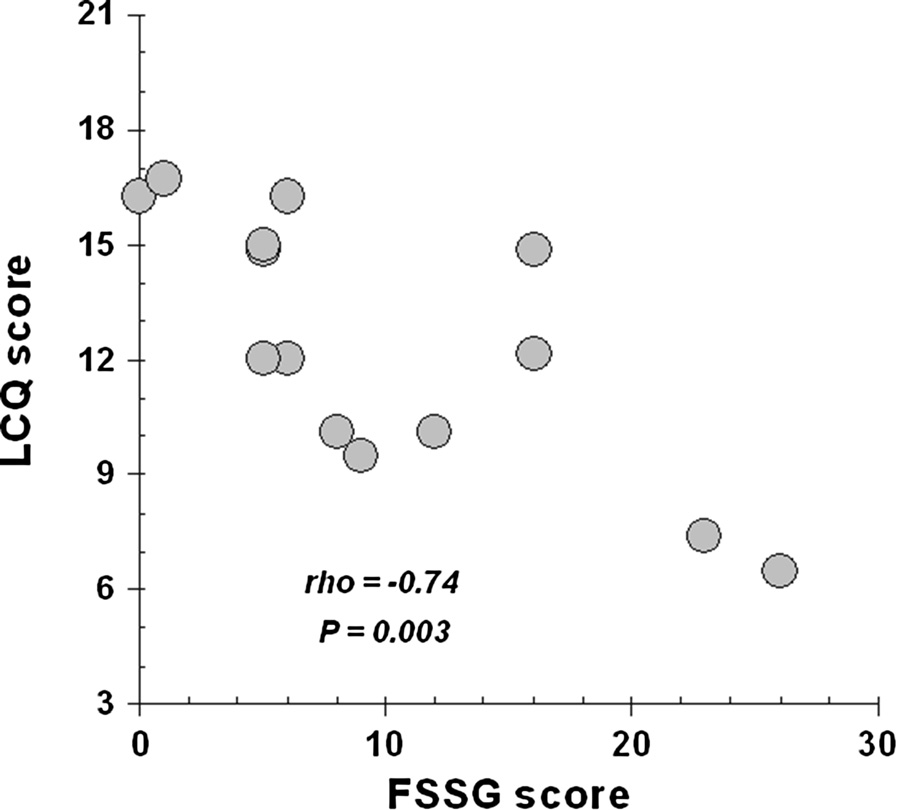
Inverse correlation between LCQ and FSSG scores.

**Figure 3:**
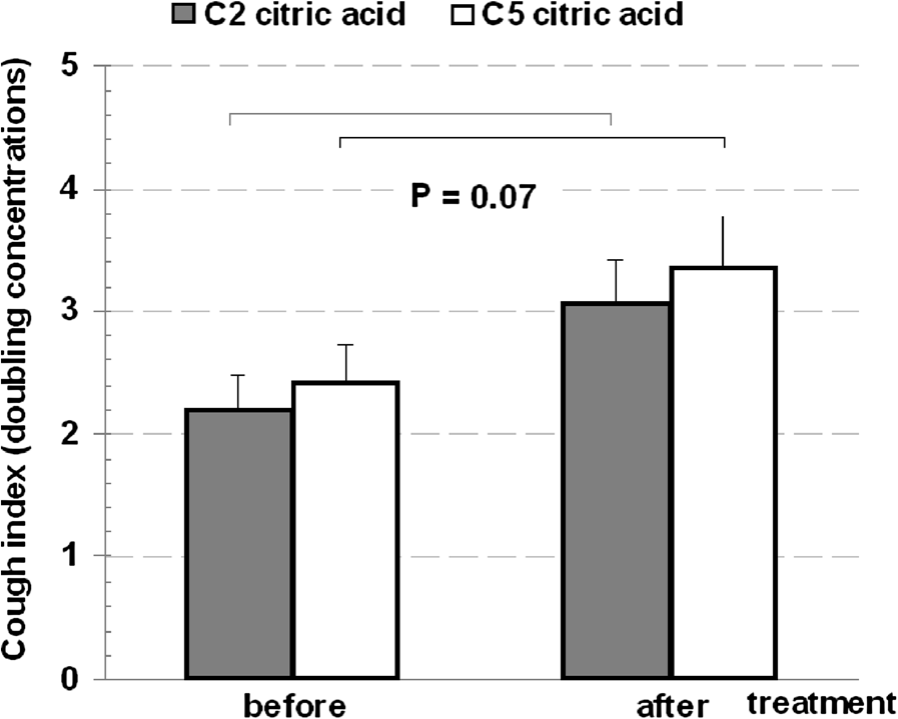
Cough indices for citric acid before and after treatment.

**Figure 4:**
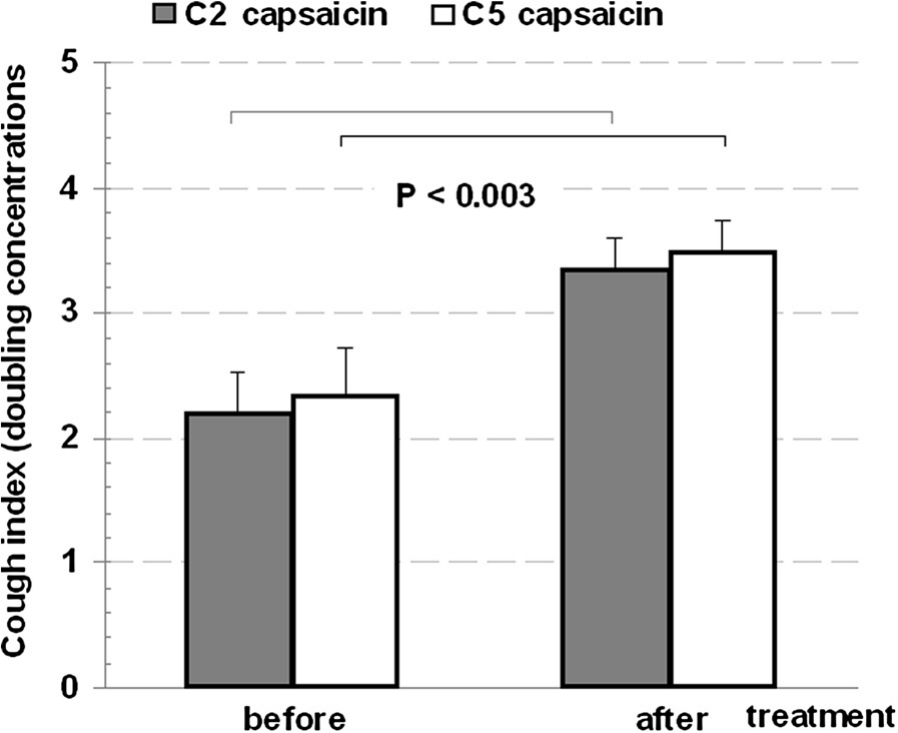
Cough indices for capsaicin before and after treatment.

Interestingly, there was a highly significant inverse correlation between initial LCQ scores and GER scores: R = −0.73, P = 0.003 (Figure 2). Lung function parameters FVC% predicted (110.6 ± 5.3 vs. 108.8 ± 6.1), FEV1% predicted (111 ± 5.1 vs. 107 ± 4.5) and EBT (34.46 ± 0.14 vs. 34.53 ± 0.12) did not change significantly after treatment. Nevertheless, cough threshold exhibited differential change for capsaicin (C_5_ more pronounced than C_2_) but for citric acid not substantial enough to reach significance (Figures 3 &4).

The results from the laboratory workup showed decrease in lactoferrin (from 9.94 ± 0.46 to 9.01 ± 0.49, p < 0.05) and ECP (from 17.33 ± 2.91 to 11.29 ± 1.70, p < 0.05) levels. However, MPO levels significantly raised (7.87 ± 0.46 vs. 8.90 ± 0.50) after treatment. Neither hsCRP (P = 0.199) nor eosinophil count (P = 0.985) altered enough to reach statistical significance.

## Discussion

Chronic cough is a burdensome health problem for patients and diagnostic and treatment challenge for the managing physicians. While cough in asthma is usually brought under control by treatment as outlined in the GINA guidelines, the treatment of UACS and GER as triggers of cough, occurring separately or concomitantly, has not been well standardized. Moreover, in both of these conditions cough can be the sole presenting symptom [26,27]. CysLTRAs have been approved for additional treatment of asthma and their effect of cough variant asthma has also been explored [16,18,19,28]. Nevertheless, the effectiveness of montelukast and other CysLTRAs has not been investigated in patients with other most frequent CC triggers. Cysteinyl-leukotrienes are pivotal inflammatory mediators which exert their biological effects through interaction with their receptors and are known to mediate plenty of pathological processes including submucosal edema, airway smooth muscle cell proliferation and contraction, mucus production, recruitment and activation of inflammatory cells, mainly eosinophils [29]. Several studies indicate elevated levels of Cys-LTs in induced sputum in different conditions causing chronic cough [30,31]. Our results indicate an effect of the CysLTRA montelukast on different markers of inflammation in the heterogeneous group of 14 consecutive patients with CC referred to our tertiary clinic.

ECP as a marker of eosinophil activation showed a decrease resulting from the treatment, which can possibly indicate that although the absolute eosinophil count is not out of the limits, eosinophil priming is still present in patients with UACS, some of them with underlying allergic rhinitis and atopy.

Lactoferrin is a multifunctional protein that among other things is present in the secondary granules of neutrophil granulocytes. Our results showed significant reduction of the lactoferrin levels which might be interpreted to be due to the ability of leukotrienes to stimulate lactoferrin release from neutrophils [32]. Hence, their blockage will prevent them from exerting this effect.

Myeloperoxidase is most abundantly expressed in neutrophils and its major role is to aid in microbial killing and facilitate neutrophilic inflammation. We observed that MPO values rose after treatment contrary to common sense expectations. Several studies have attempted to elucidate the interplay between MPO and leukotrienes [33,34]. In the light of these the increase in MPO can be explained with the role of MPO in inactivation of leukotrienes. Montelukast as a Cys-LT_1_R blocker only affects the mechanism of their action not the amounts of CysLTs. As a consequence of the receptor blockage unutilized leukotrienes could pile up and a feedback increase of MPO would be needed to inactivate them. This is an interesting interrelation that to our knowledge has not been described so far.

As opposed to the systemic biomarkers of inflammation, montelukast did not affect the outcomes of the study related to the lower airways, EBT and lung function tests. EBT is an airway inflammatory marker which reflects the thermal rise due to inflammation mainly in the intrathoracic airways. The initial values of EBT and the lung function tests were fairly uniform and did not change significantly after treatment, suggesting that our patient population was rather homogeneous and free from lower airway involvement. Indeed, only one patient had a positive methacholine test in the moderate range. Additionally, he did not present with any other symptoms characteristic for asthmatic state.

Two week therapy with montelukast selectively decreased the cough reflex sensitivity to capsaicin with high statistical significance, and while a decrease was outlined also for citric acid, it was not significant. Allegedly, montelukast lowers the inflammatory load making the cough receptors less prone to activation. The significant decrease of the cough threshold sensitivity to capsaicin could be interpreted as a much more specific effect of montelukast on the inflammatory substances implicated in CC such as histamine, bradykinin, prostaglandins and substance P than the non-specific mechanical and acidic irritants [28,35].

None of our patients had typical GER symptoms alone. It may be speculated that altered state of the tissues of laryngeal and pharyngeal region in UACS may enhance the tussigenic effects of GER supporting the notion of pathological cough reflex hypersensitivity [22].

In our study we made use of a real-life design to test the potential of a well-known therapeutic agent with good safety profile to help patients with CC. It can only be viewed as preliminary proof-of-concept to be further elaborated by blinded controlled trials. We realize that our pilot study has limitations, mostly related to its sample size and lack of “gold standard” procedures for diagnostic confirmation. The starting point of the approach we assumed was that in real life physicians rarely have the time and resources to carry out all investigations to confirm or to rule out the separate options. We thought of montelukast as a safe means to try out in CC patients without firm signs of undisputed pathology like asthma, UACS, GERD or other organic causes. If negative within the time span used in this study, this strategy should have been substituted by further diagnostic/therapeutic attempts. It was an attempt to confirm an initial hypothesis for local and systemic involvement of cysteinyl- leukotrienes in different configurations of CC. As montelukast is applied through the oral route and has systemic anti-inflammatory effects, it may have advantages over routinely prescribed antitussive drugs without such activity. If the leading bothersome symptom of cough is suppressed in the treated patients, the further elucidation and management of the condition should continue along the same line basically pointing to UACS alone or with superimposed features of GER. Thus montelukast could be a valuable therapeutic alternative with proven safety profile and overall cost-effectiveness. A different study design is needed to assess the optimal duration of montelukast treatment and the sustainability of its therapeutic effect.

## Conclusion

In conclusion our pilot study supports the feasibility of montelukast as initial exploratory treatment of patients with chronic cough in the primary care setting. This could be a new probationary approach, but its benefits and potential pitfalls need to be further evaluated.

## Abbreviations

CC: Chronic cough; EBT: Exhaled breath temperature; ECP: Eosinophil cationic protein; FSSG: Scale for the symptoms of GERD; GERD: Gastro-esophageal reflux disease; LCQ: Leicester Cough Questionnaire; LTRA: Leukotriene receptor antagonist; MPO: Myeloperoxidase; UACS: Upper airway cough syndrome.

## Competing interests

All authors agreed with the final draft of the manuscript and they have no competing interests.

## Authors’ contributions

RM recruited the patients, performed measurements, including all cough measurements, and prepared the manuscript, TK and DN recruited patients and performed measurements, MR, ZD, MS, VP recruited patients, KB performed all laboratory measurements, VD and PP did the critical revision of the manuscript, TP conceived and designed the study, did the analysis and interpretation of the data, critically revised the manuscript and coordinated the whole project. All authors read and approved the final manuscript.
